# Production of amphiregulin and recovery from influenza is greater in males than females

**DOI:** 10.1186/s13293-018-0184-8

**Published:** 2018-07-17

**Authors:** Meghan S. Vermillion, Rebecca L. Ursin, Denise I. T. Kuok, Landon G. vom Steeg, Nicholas Wohlgemuth, Olivia J. Hall, Ashley L. Fink, Eric Sasse, Andrew Nelson, Roland Ndeh, Sharon McGrath-Morrow, Wayne Mitzner, Michael C. W. Chan, Andrew Pekosz, Sabra L. Klein

**Affiliations:** 1 W. Harry Feinstone Department of Molecular Microbiology and Immunology, The Johns Hopkins Bloomberg School of Public Health, Baltimore, MD USA; 2 Molecular and Comparative Pathobiology, Johns Hopkins School of Medicine, Baltimore, MD USA; 3 Department of Biochemistry and Molecular Biology, The Johns Hopkins Bloomberg School of Public Health, Baltimore, MD USA; 4 School of Public Health, LKS Faculty of Medicine, The University of Hong Kong, Pokfulam, Hong Kong SAR China; 5 Department of Environmental Health and Engineering, The Johns Hopkins Bloomberg School of Public Health, Baltimore, MD USA; 6 Department of Pediatrics, The Johns Hopkins School of Medicine, Baltimore, MD USA

**Keywords:** Epidermal growth factor, H1N1, Inflammation, Resilience, Tolerance, Testosterone

## Abstract

**Background:**

Amphiregulin (AREG) is an epidermal growth factor that is a significant mediator of tissue repair at mucosal sites, including in the lungs during influenza A virus (IAV) infection. Previous research illustrates that males of reproductive ages experience less severe disease and recover faster than females following infection with IAV.

**Methods:**

Whether males and females differentially produce and utilize AREG for pulmonary repair after IAV infection was investigated using murine models on a C57BL/6 background and primary mouse and human epithelial cell culture systems.

**Results:**

Following sublethal infection with 2009 H1N1 IAV, adult female mice experienced greater morbidity and pulmonary inflammation during the acute phase of infection as well as worse pulmonary function during the recovery phase of infection than males, despite having similar virus clearance kinetics. As compared with females, AREG expression was greater in the lungs of male mice as well as in primary respiratory epithelial cells derived from mouse and human male donors, in response to H1N1 IAVs. Internalization of the epidermal growth factor receptor (EGFR) was also greater in respiratory epithelial cells derived from male than female mice. IAV infection of *Areg* knock-out (*Areg*^−/−^) mice eliminated sex differences in IAV pathogenesis, with a more significant role for AREG in infection of male compared to female mice. Deletion of *Areg* had no effect on virus replication kinetics in either sex. Gonadectomy and treatment of either wild-type or *Areg*^−/−^ males with testosterone improved the outcome of IAV as compared with their placebo-treated conspecifics.

**Conclusions:**

Taken together, these data show that elevated levels of testosterone and AREG, either independently or in combination, improve resilience (i.e., repair and recovery of damaged tissue) and contribute to better influenza outcomes in males compared with females.

## Background

Sex differences are reported in the severity and outcomes of many pulmonary diseases. For example, after puberty and into adulthood, human females are at greater risk of developing allergy-induced asthma, chronic bronchitis, chronic obstructive pulmonary disease (even among non-smokers), and have more severe cystic fibrosis than their male counterparts [1]. In adult mice, pulmonary infection with *Psudomonas aeruginosa* causes a worse outcome in females than males, with females exhibiting a higher bacterial burden, greater inflammation, and higher concentrations of TNF-α than males [2]. In contrast, respiratory diseases caused by infection with either *Streptococcus pneumonia* or *Bacillus anthracis* results in a worse outcome in adult male compared with female mice, with males having greater bacterial burden and inflammation in their lungs [2, 3]. There is also evidence that hospitalization rates and severity of disease following infection with *Mycobacterium tuberculosis* are greater for human males than females, but the contributions of biological sex versus gender-associated factors are debated [4–6]. In most cases of infectious or inflammatory diseases in the respiratory tract, the etiology of the sex-specific differences in disease outcome is not known.

We have studied the complexities of sex differences in pulmonary inflammation in the context of influenza pathogenesis using small animal models and primary cell cultures derived from humans and mice [7–12]. Using murine models, others and we have shown that females develop higher pulmonary inflammatory responses and experience a more severe outcome from influenza A virus (IAV) infection than males, despite the sexes having comparable virus titers [7, 8, 13–15]. Acute infection with IAVs causes clinical illness in male and female mice, leading to a transient reduction in testosterone during the acute phase of infection [12] and a more persistent reduction in circulating estradiol and progesterone, with a loss of reproductive function in females [7]. In males, testosterone protects and castration exacerbates pulmonary inflammation during IAV infection, without affecting virus titers [12]. Treatment with either estradiol or progesterone protects females against infection-induced morbidity and mortality from IAV infection [7, 9, 11, 16, 17]. Treatment of female mice with estradiol appears to protect against IAV infection by dampening the inflammatory responses associated with tissue damage and promoting higher antibody responses to influenza vaccination [7, 9, 16, 17]. Progesterone, on the other hand, promotes repair of damaged tissue following IAV infection, including production of the epidermal growth factor-like molecule, amphiregulin (AREG) [11].

Repair of the damaged lung tissue following IAV infection is generally orchestrated by both immune cells (e.g., regulatory T cells and macrophages) and epithelial cells and involves the production of cytokines and growth factors [18, 19]. In response to damage, epithelial cells release factors, including AREG, that can promote repair and integrity of lung tissue damaged during IAV infection [20]. AREG is not only predominantly expressed in epithelial cells but also can be expressed in activated immune cells [21]. AREG is constitutively expressed during development and in homeostatic states but increases dramatically in response to inflammation or infection. The effects of AREG are mediated by its low affinity binding to the epidermal growth factor receptor (EGFR) [22]. The unique activation of EGFR by AREG is hypothesized to aid in tissue repair through differentiation and proliferation of tissue resident cells, such as epithelial cells, which express EGFR [23]. Whether there are sex-dependent differences in AREG responses, however, has not been reported because studies either use only female mice or do not report the sex of the mice [20, 24–29]. Because males recover from IAV infection faster than females, we hypothesized that production of AREG and subsequent repair of damaged pulmonary tissue may be greater in males than in females and may be dependent on testosterone.

## Methods

### Animals

Adult (7–9 weeks old) male and female C57BL/6 mice were purchased from Charles River Laboratories (Frederick, MD). *Areg*^+/−^ (C57BL/6 129 Sv) mice were kindly provided by Dr. Marco Conti (University of California San Francisco) and bred to obtain *Areg*^*−/−*^ and *Areg*^*+/+*^ littermates. Mice were housed five per microisolator cages under standard BSL-2 housing condition with food and water ad libitum. All animal procedures were approved by the Johns Hopkins University Animal Care and Use Committee under animal protocol M015H236.

### Cell cultures

Primary human alveolar epithelial cells (type I-like pneumocytes) were isolated from non-malignant human lung tissues as previously described [30]. Briefly, human lung tissues were cut into pieces of < 0.5 cm and digested with 0.5% trypsin (Gibco, Invitrogen) and 4 U/ml elastase (Worthington Biochemical Corp). After digestion was finished, the macrophages were depleted from the cell suspension using CD14 antibodies and the remaining epithelial cells were cultured in a tissue culture flask using supplemented small airway basal medium (SABM) (Lonza).

Primary differentiated murine tracheal epithelial cell (mTEC) culture conditions and media were generated as previously described [11, 31]. Briefly, epithelial progenitor cells isolated from tracheal tissue were grown in collagen-coated 24-well Transwell inserts until a consistent high transelectrical reading (TER) was reached. The cultures were then exposed to an air-liquid interface by removing the apical media and incubated at 37 °C for 12–14 days before use.

### Virus infection, quantification, and purification

For animal studies, mice were infected with the mouse-adapted influenza A virus, A/California/04/09 (ma2009; H1N1) generated by Dr. Andrew Pekosz from a published sequence [32]. For the infections, mice were anesthetized with a ketamine (80 mg/kg) and xylazine (8 mg/kg) cocktail and inoculated intranasally with 30 μl of a low dose of ma2009 virus (0.04 mouse lethal dose [MLD_50_]) or mock-infected with DMEM alone. Mice were monitored daily for changes in body mass and rectal temperature.

For virus quantification in the lungs, the 50% tissue culture infectious dose (TCID_50_) was determined. Ten-fold serial dilutions of lung homogenates were plated onto monolayers of Madin-Darby canine kidney (MDCK) cells in replicates of 6 for 6 days at 32 °C. Cells were stained with naphthol blue black (Sigma Aldrich) and scored for cytopathic effects. The TCID_50_ titer was calculated according to the Reed-Muench method.

For mTEC culture infections [33, 34], the apical surface was washed twice with PBS, followed by incubation with approximately 3 × 10^7^ TCID_50_ units of 2009 H1N1 diluted in 100 μl DMEM media for 1 h at 37 °C, resulting in a multiplicity of infection (MOI) of approximately 10 infectious units per cell. The inoculum was removed, the apical surface washed three times with PBS, and the cells were incubated at 37 °C. At the indicated times post-infection, 150 μl of DMEM was added to the apical surface and the cells were incubated at 37 °C for 5 min. The apical wash and the basolateral media were then removed and stored at − 70 °C. The basolateral media was replaced with fresh media, and the cultures were returned to 37 °C. Infectious virus titers in the apical supernatant were determined by TCID_50_ assay.

For human cell culture studies, the IAV used was a seasonal H1N1 virus, A/Hong Kong/54/98, isolated from humans and passaged in MDCK cells. Virus titer was determined by using a TCID_50_ assay in MDCK cells in the presence of 1 μg/ml of tosylsulfonyl phenylalanylchloromethyl ketone (TPCK)-treated trypsin (Sigma). Primary human alveolar epithelial cells were infected with H1N1 IAV at an MOI of 2. The virus inoculum was removed after 1 h of virus incubation at 37 °C and 5% CO_2_. The cells were then washed with PBS and replenished with culture medium.

### Gonadectomy and testosterone administration

Young adult male mice were anesthetized with ketamine/xylazine cocktail and bilaterally gonadectomized as previously described [7, 8, 12]. All animals were given 2 weeks to recover from surgery prior to testosterone treatment. Testosterone was administered by subcutaneously implanting a silicone capsule (0.040 mm in inner diameter, 0.085 mm in outer diameter, 7.5 mm length) containing 100% crystalline testosterone propionate (Sigma) between the scapulae. The capsules were equilibrated in sterile physiological saline for 12 h at 37 °C prior to implantation. Animals in the placebo group received implants of empty capsules. All males were treated with testosterone for 1 week prior to infection.

### Histopathology and immunohistochemistry in lung sections

Lungs were inflated at constant pressure of 25 cmH_2_0, fixed in zinc-buffered formalin (Z-fix, Anatech). Tissues were embedded in paraffin, cut into 5 μm sections, and mounted on glass slides. Slides were stained with hematoxylin and eosin (H&E) and used to evaluate lung inflammation. Histopathological scoring was performed by a single blinded observer using a 0–3 scale (0, no inflammation; 1, mild inflammation; 2, moderate inflammation; and 3, severe inflammation) for the following parameters: bronchiolitis, alveolitis, vasculitis, perivasculitis, necrosis, consolidation, and edema [11]. The sum of these parameters represents the cumulative inflammation score. The percentage of lesioned areas within each tissue section was evaluated using ImageJ software [12]. For immunofluorescence staining, slides were deparafinized with xylene and rehydrated in graded ethanol. Heat-induced antigen retrieval with citrate buffer was performed, and slides were blocked with 3% normal donkey serum with 0.5% BSA prior to overnight incubation with anti-AREG mouse (R&D) and monoclonal anti-β-tubulin IV (BioGenex) antibodies. Slides were then washed in PBS + 0.05% Tween-20 and stained with secondary AF555-linked donkey anti-goat (ThermoFisher) for AREG and AF488-linked donkey anti-mouse (ThermoFisher) for β-tubulin. Slides were then treated against autofluorescence using 0.3% Sudan Black B (Sigma) diluted in 70% ethanol and mounted using anti-fade medium containing DAPI (ProLong Gold from Cell Signaling Techonology). Images were taken using a Nikon Eclipse E800 (for H&E) or a Zeiss AxioImager M2 (for immunofluorescence) and analyzed using ImageJ (NIH).

### mTEC culture immunofluorescence and confocal microscopy

The mTEC cultures were harvested at 18 hpi. The cultures were washed twice in cold PBS, fixed with 4% paraformaldehyde in PBS for 15 min, and washed twice in cold PBS. Fixed cells were then permeabilized in 0.2% Triton-X100 (Sigma) for 10 min before being washed, blocked in PBS with 2% donkey serum and 0.5% BSA (blocking solution), stained with monoclonal anti-HA mouse antibody (BEI Resources NR-42021; diluted 1:100 in blocking solution) and polyclonal anti-EGFR rabbit antibody (ThermoFisher PA1–1110; diluted 1:200 in blocking solution) followed by secondary AF488-linked donkey anti-rabbit antibody and AF555-linked donkey anti-mouse antibody (both diluted 1:500 in blocking solution). Cells were washed after each step by dipping membrane sections in PBS containing 0.2% Tween-20 12 times. Cells were washed a final time in MilliQ water before mounting in Prolong gold antifade with DAPI (ThermoFisher). Cells were imaged using a Zeiss LSM 700 microscopy with Zen software for image acquisition. Images were taken with a × 63 objective in Z-stack mode with 0.5 μm optical sections. Images were analyzed and processed for publication with Volocity and FIJI.

### Real-time reverse-transcription PCR

Snap-frozen lung tissues or mTECs were homogenized in TRIzol, and RNA was purified by chloroform extraction. RNA concentration and purity was measured using a NanoDrop (ThermoFisher Scientific). The RNA concentration in each sample was standardized to 1 μg using RNAse-free water. Reverse transcription was carried out using the iScript cDNA synthesis kit (Biorad) according to the manufacturer’s protocol. Pre-designed *Areg* (Mm.PT58.31037760) and *Gapdh* (Mm.PT.39a.1) PrimeTime Primers were purchased from Integrated DNA Technologies. Semi-quantitative RT-PCR was performed in 96-well optical reaction plates using the SsoFast EvaGreen Supermix (Biorad) on the StepOnePlus RT-PCR system (Applied Biosystems). Gene expression was normalized to *Gapdh* (ΔCt) and/or mock-infected samples (ΔΔCt).

For human cells, 24-h post-infection, total RNA of the alveolar epithelial cell lysates was extracted using the RNeasy Mini Kit (Takara Bio Inc.) according to the manufacturer’s instructions. cDNA was produced using the PrimeScript RT Reagent Kit (Takara Bio Inc.) according to manufacturer’s instructions. The mRNA expression of target genes was accessed by SYBR Premix Ex Taq II kit (Takara Bio Inc.) according to the manufacturer’s instructions and quantified using ABI ViiA 7 Real-Time PCR System (Applied Biosystems). The cycle threshold (Ct) values or amplification curves data were converted to the relative gene copy number, and each target gene expression was normalized to the housekeeping beta-actin gene expression. Primer sequences for detection of influenza virus matrix gene (M gene) and beta-actin were described previously [30]. Human amphiregulin gene forward primer 3′-ACCTACTCTGGGAAGCGTGA-5′ and reverse primer 3′-AGCCAGGTATTTGTGGTTCG-5′ were used. Gene expression was normalized to *Gapdh* and mock-infected samples (ΔΔCt).

### Pulmonary function phenotyping

Pulmonary function analyses were performed 14 days post-infection (dpi). Respiratory rate, tidal volume, and minute ventilation were determined by barometric whole-body plethysmography performed on unanesthetized mice, as previously described [35]. Mice were then anesthetized with a ketamine/xylazine cocktail (100 mg/kg and 10 mg/kg, respectively), and a tracheotomy was performed for cannulation with an 18-g stub needle. 0.8 ml of a gas mixture containing 0.3% neon, 0.3% CO in room air was quickly injected through the cannula into the lungs, held for 9 s, then quickly withdrawn for measurement of lung diffusion capacity (DF_CO_) by gas chromatography (Inficon, Micro GC model 3000A), as previously described [36]. The DF_CO_ for each mouse was calculated as 1 − (CO9/COc)/(Ne_9_/Ne_c_) (c = calibration gas, 9 = gas from the 9s exhaled sample). Following measurement of DF_CO_, mice were mechanically ventilated with 100% oxygen using a Flexivent system (Scireq) at a rate of 150 breaths/min and a tidal volume of 10 ml/kg with a PEEP of 3 cm H_2_O. Respiratory resistance (Rrs) and compliance (Crs) were measured 1 min following a deep inspiration to 30 cmH_2_O.

### Statistical analyses

Morbidity data were analyzed with MANOVAs followed by planned comparisons. Virus titers, amphiregulin, histopathological, and pulmonary function data were analyzed using two-way ANOVAs or *t* tests, and significant interactions were further analyzed using the Bonferroni method for pairwise multiple comparisons. Mean differences were considered statistically significant if *p* < 0.05.

## Results

### Females recover slower than males following IAV infection

**Fig. 1:**
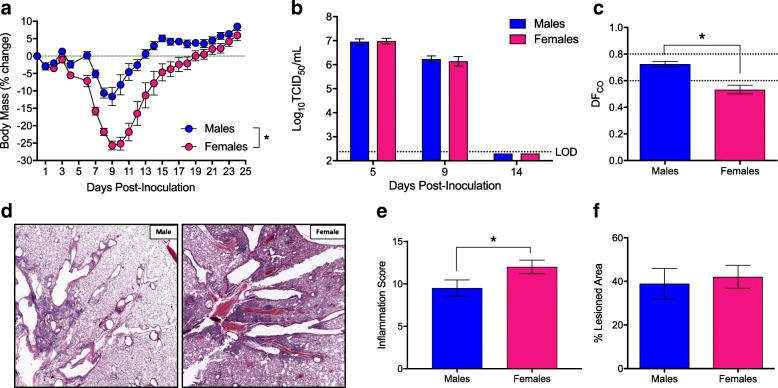
Females suffer a worse outcome than males during sub-lethal influenza A virus infection. Adult male and female mice were inoculated intranasally with a low dose of 2009 H1N1 virus and euthanized at selected time points post-challenge. Mice were monitored daily for changes in body mass for 25 days post-inoculation (dpi) (**a** *n* = 10/sex). Infectious virus titers in the lungs were measure at 5, 9, and 14 dpi (**b** *n* = 5–9/sex/time point), with the TCID_50_ limit of detection (LOD) illustrated with a dotted line. Pulmonary function, based on lung diffusing capacity (DF_CO_), was measured at 14 dpi (**c** *n* = 7–8/sex), with the dotted lines representing the values (means ± SEM) for mock-infected mice. H&E stained lung sections (**d**) collected at 14 dpi were scored for inflammation (**e** *n* = 5–7/sex) and the proportion of lung tissue damaged following infection was quantified with ImageJ (**f** *n* = 5–7/sex). Data represent means ± SEM from two independent experiments, with significant differences represented by asterisks (*)

Using historic strains of IAV, others and we have shown that female mice develop higher pulmonary inflammatory responses and experience a more severe outcome from IAV infection than males [7, 8, 13–15, 37]. To confirm and expand on previous studies, we intranasally infected adult male and female mice with a sublethal dose of the contemporary 2009 H1N1 IAV and monitored them for sex differences in morbidity. Consistent with previous reports [7, 8, 13–15, 37], females experienced greater body mass loss (*p* < 0.05; Fig. 1a), hypothermia (*p* < 0.05, data not shown), and clinical disease (*p* < 0.05; data not shown) than males. Males and females were euthanized at several time-points before (5 dpi), during (9 dpi), and after (14 dpi) peak disease to quantify pulmonary virus titers. Neither virus titers at 5 and 9 dpi nor clearance of virus by 14 dpi differed between the sexes (Fig. 1b). Although both males and females similarly clear virus from the lungs by 14 dpi, lung diffusing capacity (DF_CO_) (i.e., a marker of pulmonary function) returned to baseline faster in males than females at 14 dpi (*p* < 0.05; Fig. 1c). Lung sections were evaluated for markers of inflammation and damage during recovery (14 dpi), and females exhibited significantly greater pulmonary inflammation than males (*p* < 0.05; Fig. 1d, e), despite having similar proportions of their lungs damaged following IAV infection (Fig. 1f).

**Fig. 2:**
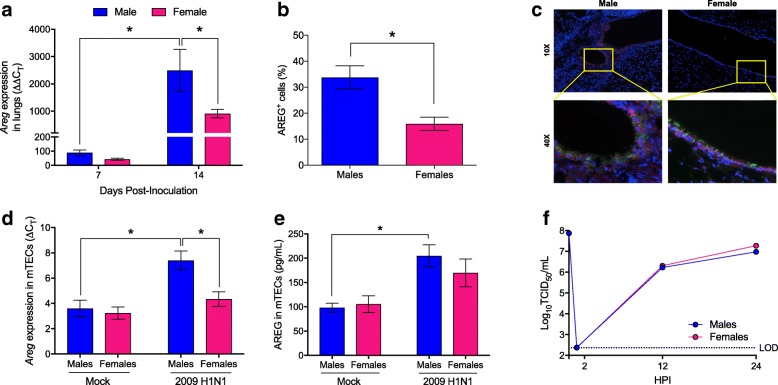
Females produce less amphiregulin (AREG) than males during influenza A virus infection. Using RNA isolated from lungs collected at either 7 dpi or 14 dpi, *Areg* mRNA expression was quantified and normalized to *Gapdh* using the ΔΔCt method (**a** *n* = 7–9/sex). Fixed lungs collected 14 dpi were used to assess AREG expression (in red) in pulmonary cells (DAPI+, in blue), and β-tubulin was used to identify ciliated epithelial cells (in green) using immunofluorescence (**b**). Representative images of bronchioles (× 10 magnification) and focused areas (× 40 magnification) are shown (**b**). The percentage of AREG+ cells in the lungs was analyzed and quantified using ImageJ (**c** *n* = 15 fields/sex). Mouse tracheal epithelial cell (mTEC) cultures were isolated and differentiated at an air liquid interface from male and female mice. Twenty-four hours after mock or 2009 H1N1 (MOI = 10) infection, *Areg* mRNA relative to *Gapdh* expression (ΔCt; **d**) and AREG (pg/ml) secretion into the basolateral media (**e**) were measured. Infectious virus titers in IAV-infected mTECs (**f**) were measured at the indicated time points (*n* = 8/sex/time point), with the dotted line indicated the TCID_50_ limit of detection (LOD). Data represent means ± SEM from two independent experiments, with significant differences represented by asterisks (*)

The epidermal growth factor, AREG, promotes proliferation of epithelial cells and protects mice from excessive pathology during IAV infection [11, 20, 38]. Analysis of *Areg* expression during IAV infection revealed greater mRNA expression during the recovery phase (i.e., 14 dpi) as compared to peak disease (i.e., 7 dpi) (*p* < 0.05; Fig. 2a). Further, both *Areg* mRNA and AREG protein were more highly expressed in the lungs of male as compared to female mice at 14 dpi (*p* < 0.05; Fig. 2a, b). Using β-tubulin as a marker for respiratory epithelial cells, AREG was shown to localize primarily in epithelial cells of the lungs (Fig. 2c).

### Respiratory epithelial cells derived from males produce more AREG than cells from females

Although AREG is produced primarily by epithelial cells [11, 39] (Fig. 2c), type 2 innate lymphoid cells (ILC2) and regulatory T cells (Tregs) produce AREG during IAV infection and contribute to pulmonary repair during the resolution of infection [20, 38–40]. To study sex differences in epithelial-specific AREG signaling in the context of IAV infection, we used an in vitro model system in which primary differentiated mTEC cultures were infected with a high MOI of 2009 H1N1 IAV. Prior to infection, no sex differences in *Areg* mRNA (Fig. 2d) or AREG protein (Fig. 2e) were observed. Twenty-four hours after infection, there was a significant increase in *Areg* mRNA (*p* < 0.05; Fig. 2d) and secreted protein (*p* < 0.05; Fig. 2e) from mTECs derived from male, but not female mice (*p* < 0.05 for interaction, Fig. 2d), and *Areg* expression was significantly greater in IAV-infected cultures from males as compared with IAV-infected cultures from females. Similar to the in vivo studies (Fig. 1b), there was no difference in the virus replication kinetics in mTECs isolated from male or female mice (Fig. 2f).

**Fig. 3:**
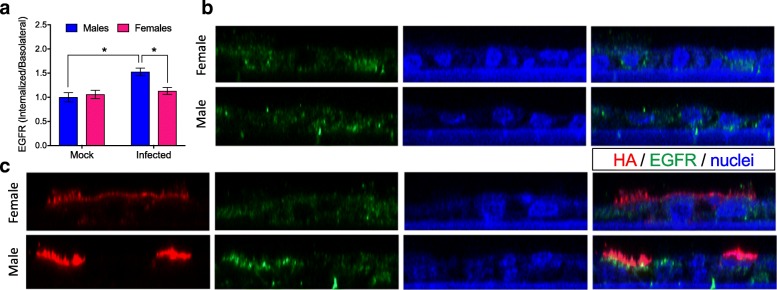
Influenza A virus infection of mouse respiratory epithelial cells results in greater internalization of the epidermal growth factor receptor (EGFR) in cells derived from males. Mouse tracheal epithelial cell (mTEC) cultures were isolated from male and female mice and either mock-infected or infected with 2009 H1N1 at a high multiplicity of infection (MOI = 10). At 24 hpi, the cultures were fixed and immunostained for the 2009 H1N1 hemagglutinin protein (HA; red). The cultures were then permeabilized and immunostained for the EGFR (green) with a nuclear counterstain (DAPI; blue). **a** Quantitation of the ratio of internalized to basolateral EGFR fluorescence signal present in panels **b** and **c**. Reconstructed Z sections from 0.5 μm optical sections obtained via confocal microscopy showing the localization of HA and EGFR in mock (**b**) or IAV-infected (**c**) mTEC cultures derived from male or female mice. The images were obtained on an LSM 700 using a × 63 objective and processed using Volocity and FIJI software. Data represent means ± SEM from two independent experiments, with significant differences represented by asterisks (*). At least eight fields were counted per condition, per experiment

AREG binds to the EGFR to have its reparative effects. Utilizing mock and 2009 H1N1-infected mTECs, we assessed whether mTECs express EGFR at the apical or basolateral plasma membranes and whether EGFR is internalized (i.e., activated [41]) to a greater extent in cultures derived from male than female mice using immunofluorescence confocal microscopy. Under mock infection conditions, mTECs expressed EGFR in a diffuse pattern that was predominately near the basolateral surface, and receptor distribution was similar between males and females (Fig. 3a, b). Following H1N1 IAV infection, EGFR localization was significantly altered in mTEC cultures derived from males, but not females (*p* < 0.05 for interaction; Fig. 3a), with a strong signal detected closer to the apical membrane of infected cells (*p* < 0.05; Fig. 3a, c). The EGFR was not localized at the apical plasma membrane, as it did not colocalize significantly with the HA protein, which is an integral membrane protein that targets to the apical membranes. The EGFR localization was consistent with its presence in endosomes, which is known to occur after binding of AREG to EGFR. These data suggest that EGFR localization changes significantly in IAV-infected mTEC cultures derived from males compared with females.

**Fig. 4:**
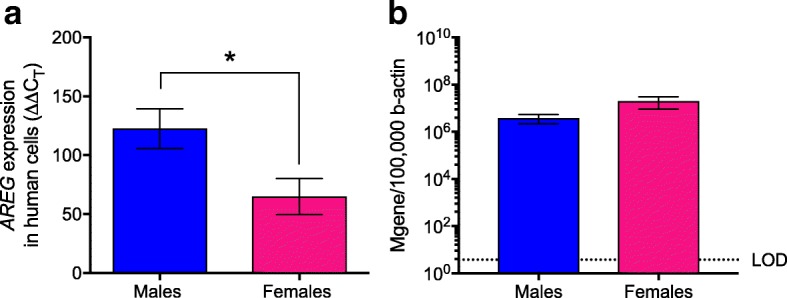
Amphiregulin (*Areg*) expression is greater in respiratory epithelial cells derived from human male than female donors following seasonal influenza A virus infection. Primary human type II alveolar epithelial lung cells were isolated from male and female donors and either mock-infected or infected with a seasonal H1N1 (A/HK/54/98) at a high MOI (MOI = 5). Amphiregulin mRNA expression was measured 24 hpi in cells derived from female (*n* = 6) and male (*n* = 4) donors (**a**). Virus levels were measured using the number of M-gene copies normalized to β-actin in cells derived from female (*n* = 6) and male (*n* = 4) donors (**b**). *Areg* mRNA expression was normalized to *Gapdh* and to mock-infected controls using the ΔΔCt method. Data represent means ± SEM from two independent experiments, with significant differences represented by asterisks (*)

To determine whether sex differences in the production of AREG in response to IAV infection are conserved across other mammalian hosts and IAV strains, primary human alveolar epithelial cells (i.e., type I-like pneumocytes) were isolated and differentiated from human lung tissues and infected with a seasonal H1N1 virus at a high MOI. Twenty-four hours post-infection (hpi), alveolar epithelial cells derived from males had higher levels of *Areg* mRNA than cells isolated from females following infection with a seasonal H1N1 virus (*p* < 0.05; Fig. 4a), despite having similar levels of viral RNA (Fig. 4b).

### Deletion of amphiregulin eliminates sex differences in IAV pathogenesis

**Fig. 5:**
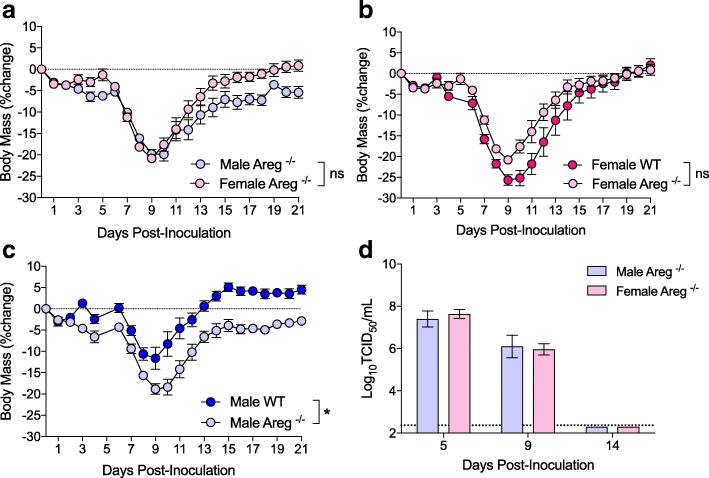
Deletion of amphiregulin (*Areg*) eliminates sex differences in influenza A virus pathogenesis. Adult male and female *Areg*^−/−^ and wild-type (*Areg*^+/+^) mice were inoculated intranasally with a low dose of 2009 H1N1 virus and euthanized at selected time points post-challenge. *Areg*^−/−^ mice were monitored daily for changes in body mass for 21 days post-inoculation (dpi) (**a** *n* = 18–19/sex) and compared with their wild-type female (**b**) and male (**c**) counterparts (*n* = 10–19/sex/genotype). Infectious virus titers in the lungs of *Areg*^*−/−*^ mice were measure at 5, 9, and 14 dpi (**d** *n* = 5–7/sex/time point), with the dotted line indicated the limit of detection (LOD) for the assay. Data represent means ± SEM from two independent experiments and significant differences are represented by asterisks (*)

If sex differences in the pathogenesis of influenza are caused by differential production of AREG, then deletion of *Areg* should eliminate sex differences in disease. To test this hypothesis, male and female *Areg*^−/−^ and wild-type (*Areg*^+/+^) mice were intranasally infected with a sub-lethal dose of 2009 H1N1 IAV and monitored for morbidity or euthanized at select dpi to evaluate pulmonary virus titers, inflammation, and pulmonary function. Consistent with our hypothesis, depletion of *Areg* resulted in similar body mass loss (Fig. 5a), hypothermia (data not shown), and clinical disease (data not shown) in males and females. The lack of a sex-differential effect in IAV pathogenesis was primarily caused by the effects of depleting *Areg* in males, as female *Areg*^−/−^ and wild-type (*Areg*^+/+^) had similar levels of morbidity (Fig. 5b), whereas male *Areg*^−/−^ mice experience greater morbidity than wild-type mice (*p* < 0.05; Fig. 5c). There was no effect of *Areg* deletion on virus replication kinetics, as both male and female *Areg*^−/−^ mice had similar peak titers and clear virus from their lungs by 14 dpi (Fig. 5d).

**Fig. 6:**
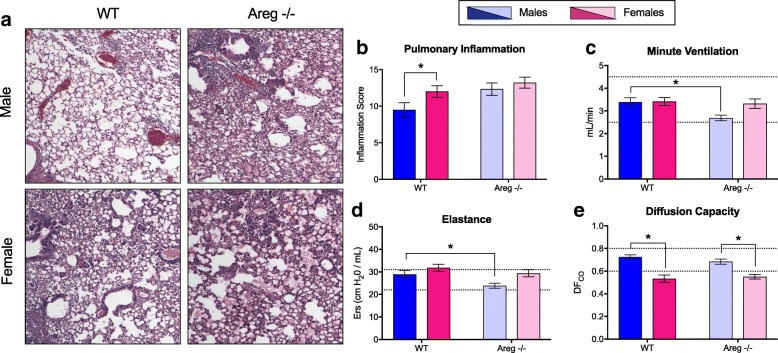
Deletion of amphiregulin (*Areg*) causes greater pulmonary inflammation and reduced pulmonary function in males during H1N1 influenza virus infection. Adult male and female wild-type (*Areg*^+/+^) and *Areg*^−/−^ mice were inoculated intranasally with a low dose of 2009 H1N1 virus and euthanized at 14 days post-inoculation (dpi; *n* = 5–7/sex/time-point). H&E stained lung sections (**a**) were scored for inflammation (**b** *n* = 6–9/sex/genotype). Pulmonary function tests were performed at 14 dpi and minute ventilation (mL/min; **c**), elastance (Rrs; **d**), and lung diffusing capacity (DF_CO_; **e**) were measured. The dotted lines represent the value (means ± SEM) for mock-infected mice and bars and represent means ± SEM from two independent experiments, with significant differences represented by asterisks (*)

Lung sections were evaluated for markers of inflammation and damage during recovery (14 dpi) in both *Areg*^−/−^ and wild-type male and female mice. Although pulmonary inflammation scores were similar between male and female *Areg*^−/−^ mice, inflammation, including alveolar inflammation and edema, was significantly greater in *Areg*^−/−^ males compared with the wild-type males (*p* < 0.05; Fig. 6a, b). In contrast, inflammation was equally high in *Areg*^−/−^ and wild-type females (Fig. 6a, b). Similarly, deletion of *Areg* had a greater effect on pulmonary function in males than females, as minute ventilation and elastance (i.e., a measure of lung recoil following expansion) were significantly reduced in male *Areg*^−/−^ as compared with the wild-type males (*p* < 0.05; Fig. 6c, d). By contrast, the one measure of pulmonary function that was not affected by deletion of *Areg* was lung diffusing capacity, which was consistently lower in females than males, regardless of the presence or absence of *Areg* (*p* < 0.05; Fig. 6e).

### Testosterone-mediated protection against IAV is independent of AREG

**Fig. 7:**
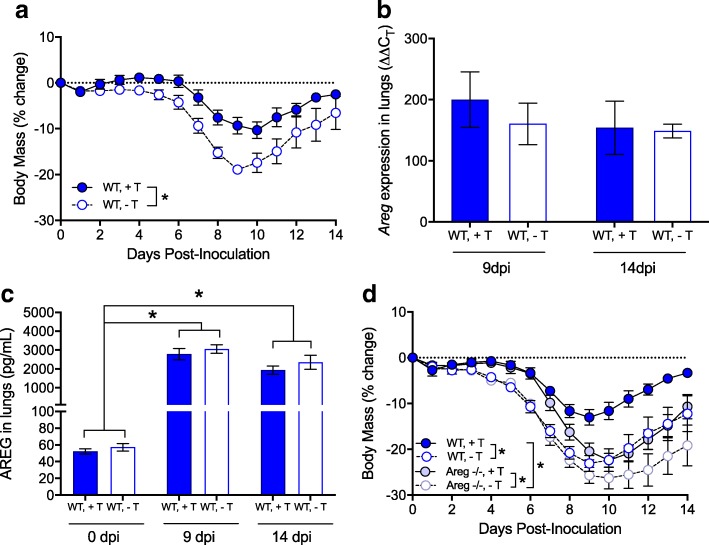
Testosterone and amphiregulin (AREG) independently protect against influenza A virus disease in males. Adult male wild-type mice (*n* = 10/treatment) were gonadectomized, treated with either placebo (Gdx) or testosterone (Gdx + T), inoculated intranasally with a low dose of 2009 H1N1 virus, monitored for changes in body mass (**a**), and euthanized at one of several time points to measure *Areg* mRNA (ΔΔCt; **b**) and protein (**c**) in the lungs. Adult male wild-type (*Areg*^*+/+*^) and *Areg*^−/−^ mice (*n* = 12–15/treatment/genotype) were gonadectomized, treated with either placebo (−T) or testosterone (+T), infected with 2009 H1N1 virus, and monitored for changes in body mass for 14 days post-inoculation (dpi; **d**). Data represent means ± SEM from two independent experiments, and significant differences are represented by asterisks (*)

In the reproductive tract, androgens regulate the expression of AREG [42, 43]. To test whether AREG-mediated protection in males was testosterone-dependent, wild-type male mice were gonadectomized and treated with either testosterone or placebo, infected with 2009 H1N1, monitored for morbidity, and euthanized at several times post-infection. Consistent with previous studies [12], gonadectomized males treated with testosterone exhibited less morbidity than males treated with placebo (*p* < 0.05; Fig. 7a). In contrast to our hypothesis, the presence or absence of testosterone did not affect either *Areg* expression (Fig. 7b) or secreted protein (Fig. 7c) in the lungs of male mice. Thus, AREG production was induced during IAV infection, regardless of testosterone treatment (*p* < 0.05; Fig. 7c). Furthermore, treatment of either gonadectomized wild-type (*Areg*^+/+^) or *Areg*^−/−^ males with testosterone improved the outcome of IAV relative to their placebo-treated male counterparts (*p* < 0.05; Fig. 7d). Taken together, these data illustrate that AREG-mediated protection against severe IAV does not require the presence of testosterone.

## Discussion

Sex differences in the pathogenesis of IAVs in humans are reported for pandemic and avian strains and to a lesser extent for seasonal strains [44]. Mouse models have been integral for supporting and expanding the epidemiological data by illustrating that across diverse strains of mice (e.g., BALB/c and several C57BL/6 substrains and crosses) infected with diverse IAVs, including H1N1, H3N1, H3N2, and H7N9, adult females experience more inflammation and suffer a more severe course of disease than adult males, despite having similar virus replication kinetics in the lungs [7, 8, 13, 15, 37]. Consistent with these previous studies, females infected with 2009 H1N1 experienced great pulmonary inflammation and were slower to recover from infection than males, despite having similar kinetics of virus replication and clearance from the lungs. Together, these data suggest that the failure to resolve inflammation as opposed to an inability to control virus replication contributes to the worse outcome from IAV infection in females compared with males.

In addition to limiting pulmonary inflammation, males repaired pulmonary tissue faster than females as illustrated by the improved oxygen exchange in the lungs after virus clearance in males. Male-biased repair of wounded mucosal tissues, including the lungs and oral cavity, has been reported in humans and mice [45, 46]. In each case, reduced inflammation in mucosal tissues from males is associated with better tissue recovery in males compared with females. In contrast, healing of dermal wounds is faster for females than males, with estrogens dampening local inflammation and cellular infiltrates to expedite repair of wounded tissue [47, 48], and testosterone or dihydrotestosterone having deleterious effects on the repair of wounded tissue [48]. The tissue-specific mechanisms mediating repair of mucosal versus dermal tissue may contribute to disparate findings between the sexes. In pulmonary tissue, resolution of inflammation and repair of damaged tissue is faster for males than females following either injury [46] or infection (this study).

Wound repair and recovery of mucosal tissue following injury or infection are mediated by the production of cytokines and growth factors in both immune and epithelial cells [18, 19]. Damaged epithelial cells release factors, including AREG, that can promote repair and integrity of lung tissue damaged during IAV infection [20]. Expression of AREG was greater in the lung tissue as well as in respiratory epithelial cells derived from males as compared with females during IAV. The sex differential expression of *Areg* was greater in the lungs than in mTECs, possibly because in addition to epithelial cells, immune cells in the lungs (e.g., CD4+ T cells and ILCs) also produce AREG [21]. Sex differences in AREG production in immune cells require consideration. Males also depended on AREG more than females for faster recovery from IAV, because when AREG was deleted from mice, the impact on pulmonary inflammation and function was significantly greater for male than for female mice. Females may still benefit from elevated AREG to repair damaged lung tissue following IAV infection, but because wild-type female mice already produce lower levels of AREG than males, eliminating AREG in the sex that already expressed low levels of AREG had little impact on the long-term pathogenesis of IAV.

Although several cell types can produce AREG, epithelial cells at mucosal sites are primary producers of AREG [21]. Respiratory epithelial cells are also the primary cell type infected with IAVs. The reparative effects of AREG are mediated by its low affinity binding to the EGFR [49, 50], which results in slower EGFR internalization and sustained downstream signaling [22]. AREG-EGFR signaling is hypothesized to aid in tissue repair through differentiation and proliferation of tissue resident cells, such as epithelial cells, which express EGFR [23]. Moreover, in cancer models, there is evidence of bidirectional cross-talk between sex steroid receptors, including androgen and progesterone receptors, and EGFR signaling pathways [51–53]. In the present study, although IAV replication in respiratory epithelial cells derived from either humans or mice did not differ between the sexes, production of AREG and internalization of EGFR was greater in respiratory epithelial cells derived from males than females, which may be mediated sex steroid hormone signaling.

Previous studies illustrate that treatment of gonadectomized wild-type female mice with progesterone significantly increases production of AREG, enhances cellular proliferation in the lungs, reduces pulmonary inflammation, and improves the outcome of IAV when compared with gonadectomized females treated with placebo [11]. In the absence of AREG (i.e., *Areg*^−/−^), progesterone does not improve the outcome of IAV in females, suggesting that the protective effects of progesterone are dependent on AREG signaling in females [11]. Progesterone treatment of mTECs also increases production of AREG following injury and promotes faster repair of the cellular monolayer, illustrating that respiratory epithelial cells are responsive to progesterone and respond by increasing the production of AREG to repair damaged tissue [11].

In addition to progesterone in females [11], testosterone in males has been shown to increase production of AREG in the reproductive tract [42, 43]. In the respiratory tract, however, the elevated production of AREG in males appeared to be independent of testosterone, as gonadectomy and exogenous testosterone replacement had no effect on AREG expression in the lungs during IAV infection. Furthermore, treatment of either wild-type or *Areg*^−/−^ gonadectomized male mice with testosterone improved the outcome of IAV when compared with their placebo-treated counterparts, suggesting that testosterone and AREG confer protection possibly through independent mechanisms. Whether testosterone and AREG have synergistic effects on the repair of pulmonary tissue following IAV requires further evaluation because males that were devoid of both testosterone and AREG suffered the most severe outcome from IAV, suggesting that both factors are required for optimal recovery from IAV in males.

Previous studies illustrate that testosterone is necessary to reduce the severity of influenza in young adult male mice [12]. Testosterone treatment of male mice limits pulmonary inflammation and promotes faster recovery from IAV. In addition to testosterone, genetic variation in the Y chromosome also influences immunopathology, but not virus replication, during IAV infection in males [37]. Whether the AREG gene, which is encoded on chromosome 5, is regulated by elements on the Y chromosome in males should be considered.

## Conclusions

Resistance or the ability to reduce viral load often defines protection and reduced susceptibility to infection. Although resistance is one way to defend against infection, another mechanism, called tolerance, is to limit the damage caused by the virus or the host immune response to the virus (i.e., immunopathology) [54]. Host tolerance of the damage following viral infection presumably serves to enable the host to maintain health regardless of virus burden. In the context of IAVs, males and females are equally resistant to IAVs, but males maintain greater tolerance during infection by limiting inflammation and immunopathology, while maintaining similar viral clearance kinetics as females. The data from the current study further illustrate that in addition to having greater tolerance to IAV infection, males also have faster repair of damaged tissue than females suggesting that resilience (i.e., responses that repair damaged tissue) may also be greater in males. For several infections, in which tissue damage caused by excessive immune responses to infection results in a more severe outcome from infection (e.g., HIV, influenza, and *Staphylococcus aureus*), it is females who suffer a worse outcome [7, 55–58]. The data from the present study illustrate that males may have greater tolerance and resilience to IAVs than females, as males experience less inflammation, repair damaged tissue quicker, and recover from IAV infection faster than females, which is mediated by elevated levels of both AREG and testosterone in males.

## Abbreviations

AREG: Amphiregulin
DFco: Lung diffusion capacity
EGFR: Epidermal growth factor receptor
IAV: Influenza A virus
mTEC: Mouse tracheal epithelial cell
